# Challenges in the preclinical design and assessment of CAR-T cells

**DOI:** 10.3389/fimmu.2025.1564998

**Published:** 2025-08-08

**Authors:** Radu Tomai, Javier De Las Rivas, Bogdan Fetica, Rui Bergantim, Brankica Filipic, Zarko Gagic, Katarina Nikolic, Diana Gulei, David Kegyes, Madalina Nistor, Ximena Maria Muresan, Diana Cenariu, Richard Feder, Mariana Pavel-Tanasa, Andrei Cianga, Adrian Bogdan Tigu, Raluca Munteanu, Alina Tanase, Hermann Einsele, Ciprian Tomuleasa

**Affiliations:** ^1^ Department of Hematology - Medfuture Institute for Biomedical Research, Iuliu Haţieganu University of Medicine and Pharmacy, Cluj-Napoca, Romania; ^2^ Department of Personalized Medicine and Rare Diseases - Medfuture Institute for Biomedical Research, Iuliu Haţieganu University of Medicine and Pharmacy, Cluj-Napoca, Romania; ^3^ Bioinformatics and Functional Genomics Group, Cancer Research Center (CiC-IBMCC, CSIC/USAL), Consejo Superior de Investigaciones Cientificas (CSIC) & University of Salamanca (USAL), Salamanca, Spain; ^4^ Hematology Department, University Hospital Sao Joao Porto São João (ULS), Porto, Portugal; ^5^ i3S Instituto de Investigação e Inovação em Saúde University of Porto, Porto, Portugal; ^6^ Cancer Drug Resistance Group, Institute of Molecular Pathology and Immunology of the University of Porto (IPATIMUP), Porto, Portugal; ^7^ Clinical Hematology Department, Faculty of Medicine of the University of Porto (FMUP), Porto, Portugal; ^8^ Department of Microbiology and Immunology, Faculty of Pharmacy, University of Belgrade, Belgrade, Serbia; ^9^ Department of Pharmaceutical Chemistry, Faculty of Medicine, University of Banja Luka, Banja Luka, Bosnia and Herzegovina; ^10^ Department of Pharmaceutical Chemistry, Faculty of Pharmacy, University of Belgrade, Belgrade, Serbia; ^11^ Department of Immunology, Grigore T. Popa University of Medicine and Pharmacy, Iasi, Romania; ^12^ Department of Stem Cell Transplantation, Fundeni Clinical Institute, Bucharest, Romania; ^13^ Julius Maximilians University of Würzburg, Würzburg, Germany

**Keywords:** CAR-T cell tracking, tumor organoids, antigen escape, solid tumor immunotherapy, metabolic reprogramming, HDAC inhibitors

## Abstract

The advent of immunotherapy in the treatment of cancer has opened a new dimension in the management of this complex multifaceted disease, bringing hope to many patients whose tumors have failed to respond to conventional therapies. The adoptive T cell therapy has since been extended to the treatment of several hematologic malignancies, initially in relapsed settings and more recently at the forefront of treatment due to high response rates. Despite exciting initial results, the preclinical antitumor effects of the first long-term studies show that CAR (Chimeric Antigen Receptor)-T cells have been slow to translate to the clinical setting, with early clinical trials showing suboptimal responses. The main reasons for the limited clinical performance seemed to be related to the low activation and short persistence of CAR-T cells. Thus, began a journey to improve the initial CAR structure, leading to the development of more complex constructs, which are grouped into five CAR generations. In this review, we describe the main challenges and potential solutions for the evaluation of CAR T-cell-based therapies in the preclinical setting.

## 1 Background on CAR-T targets

The emergence of immunotherapies in cancer treatment has provided a new approach to counter challenging diseases, offering hope for many patients whose conditions remained unchanged after conventional chemotherapy. The notion of harnessing the body’s own defenses and directing them towards the disease was first proposed back in the 19^th^ century, although the mechanisms involved remained long unknown. In the second half of the 20^th^ century, immune cells have been shown to be capable of eliciting an antitumoral effect and later on, tumor infiltrating lymphocytes were successfully used in the treatment of cancers (1–3). The concept of chimeric T cell receptors was later developed when T cell receptors combined with antibody-derived variable regions were shown to induce T cell activation in a non-major histocompatibility complex (MHC) mediated manner (4, 5). This seminal discovery is what led to the development of the revolutionary immune therapy which uses the transgenic Chimeric Antigen Receptor (CAR) to direct T cells towards a desired target cell and induce activation and tumor killing. CAR-T therapy has been shown to be effective in achieving clinical response in cancer patients initially in chronic lymphocytic leukemia and follicular lymphoma with the first CD19-targeting CAR-T cell therapy eventually approved by the Food and Drug Administration (FDA) in the U.S.A. in 2017 for the treatment of pediatric and young adult B-cell acute lymphoblastic leukemia (6–8). The adoptive T cell therapy has since been expanded to the treatment of multiple hematologic malignancies starting in relapsed settings and recently moving towards the front lines of treatment due to the high response rates (1, 9, 10).

**Figure 1:**
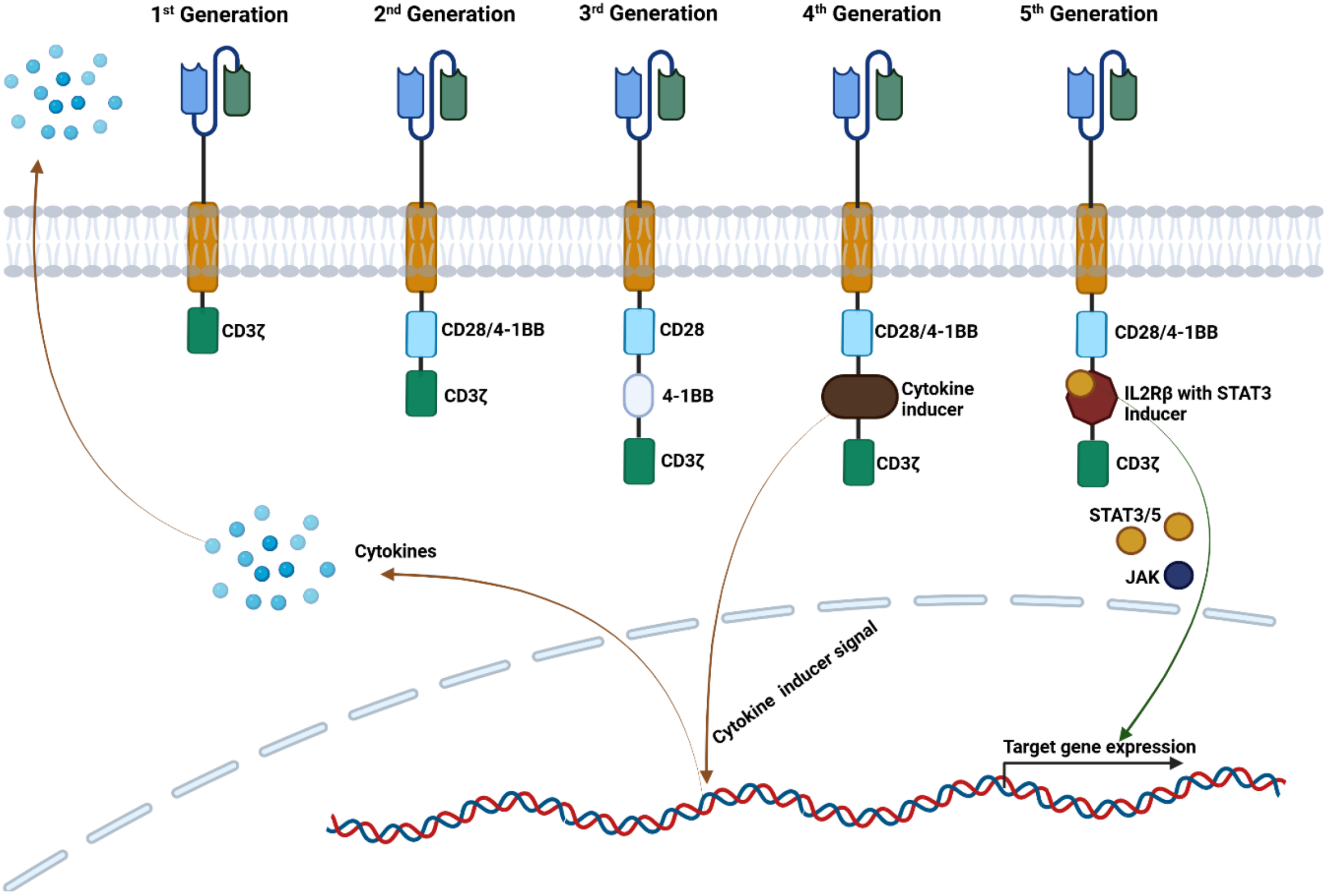
Five CAR-T cell generations. The 1^st^ Generation of CAR-T cells contain the CD3ζ domain, which triggers the intracellular signaling. The 2^nd^ Generation has a co-stimulatory domain (CD28 or 4-1BB) enhancing the cytotoxicity of the CAR-T cells. The 3^rd^ Generation has two co-stimulatory domains, offering superior cytokine secretion and CAR-T cell persistence. The 4^th^ Generation has the cytokine inducer domain, and two co-stimulatory domains, modulating cytokine secretion. The 5^th^ Generation of CAR-T cells have two co-stimulatory domains and with similar structure to the 2^nd^ Generation with a STAT3 binding site, and its activation triggers cell signaling through CD3ζ, CD28 and JAK/STAT3 signaling activating the CAR T cells and maintain proliferation.

While exciting, the preclinical antitumoral effects of initial CAR-T cells were slow to translate to the clinical setting, with early clinical trials showing suboptimal responses. Despite the remarkable initial responses observed in clinical trials, long-term outcome studies show that most of the treated patients experience progression of the disease. The main reasons for this limited success seem to be related to low CAR-T cell activation and reduced longevity/durability, as well as antigen escape. Consequently, the initial CAR structure has been continuously improved, leading to the development of more complex constructs that can be organized into five CAR generations (**Figure 1**) (1).

The first generation of CARs consists of an extracellular antibody-derived single-chain variable (scFv) region joined to CD3ζ or FcϵRIγ signal transducing endodomains by a hinge, and a transmembrane domain (11, 12). While first-generation CARs were able to induce T cell activation and produce *in vitro* and *in vivo* antitumoral effects in tumor models such as ERBB2-expressing tumors and ovarian cancer, early clinical trials employing these receptors unfortunately showed little to no tumor response and limited *in vivo* persistence of CAR T cells, varying from one to nine weeks, with longest durability in patients stimulated with interleukin 2 (IL-2) (12–17).

The second generation of CAR constructs improved on the first generation by integrating an additional costimulatory endodomain into the CAR structure to enhance function and persistence. Since normal T cell receptor (TCR)-mediated T cell activation requires costimulation, it was postulated that replicating this mechanism in CAR-T cells might enhance their activation. It was thereafter demonstrated that costimulatory signaling effectively improves CAR T cell activation regardless of whether it is exogenous, intrinsic, or originates in target cells (18–20). Costimulatory domains employed in CAR constructs originate in the activation pathways of B and T lymphocytes, where they provide the required signaling for activation. Multiple domains such as CD27, CD28, 4-1BB (CD137), OX40 (CD134) have been successfully used to augment CAR function by increasing activation, cytotoxicity and persistence (21, 22). The two most commonly used costimulatory domains are 4-1BB and CD28. While both improve efficacy, important differences have been observed among the two approaches. CD28 CAR has been shown to lead to a more robust expansion while 4-1BB CAR is associated with longer persistence, likely due to activation of non-canonical NF-kB pathway with antiapoptotic effects (23–25). It is important to note that the choice of costimulatory domain influences T cell differentiation and phenotype, though results from mouse models and clinical trials show that the efficacy between the two types of CAR is similar (24–26). Following validation of their clinical efficacy, second-generation CAR were approved for use in clinical practice, pioneered by the 4-1BB anti-CD19 CAR-T therapy in 2017 (8). Further efforts to improve CAR-T efficacy led to the development of third, fourth, and fifth generation CARs. The third-generation receptors incorporate multiple costimulatory domains, usually from different receptor families such as Ig and tumor necrosis factor superfamilies (27, 28). This approach benefits from the joint effects of each costimulatory domain such as inducible T cell costimulatory (ICOS) domain and 4-1BB, which promote persistence of CD4+ and CD8+ CAR-T cells, respectively (29). Multiple studies have shown the improved *in vivo* expansion and persistence of third generation CAR-T cells, which might prove beneficial in instances where the target antigen is scarcely expressed (30, 31). The superiority of third generation CAR-T to the second one is still to be established as, in certain instances, they underperformed compared to the second-generation. One of the proposed mechanisms for the observed lower efficacy is tonic signaling, leading to activation induced activation-induced cell death. The order of costimulatory domains on the CAR and their proximity to the cell membrane may account for this effect and might be mitigated by alternative receptor designs (32).

Digressing from the beaten path of adding new domains to the CAR receptor, the design of fourth-generation CAR-T cells aims to improve antitumor effect by secretion of cytokines to induce a proinflammatory microenvironment. In addition to the CAR, these cells, also known as TRUCKs (T cells Redirected for Antigen-Unrestricted Cytokine-initiated Killing), include a constitutive or NFAT (nuclear factor of activated T cells) inducible expression cassette. Upon CAR binding to its target antigen, CD3ζ mediatedCD3ζ-mediated phosphorylation of Nuclear factor of activated T-cells (NFAT) induces cytokine secretion, which acts to enhance CAR-T function as well as to recruit inflammatory cells (33, 34). Several cytokines known to stimulate T cell functions *in vitro* have been incorporated in TRUCKs models, with the most notable being interleukin 12 (IL-12), interleukin 18 (IL-18) and interleukin 15 (IL-15) (35, 36).

IL-12 has been reported to induce a more robust antitumor response against CD19+ positive acute leukemia and in mouse models of ovarian cancer. However, multiple studies report severe toxicity related to its potent pro-inflammatory effects and important macrophage activation (37). In one study, Il-12 TRUCKs induced significant tumor infiltration by macrophages, albeit at the expense of a decrease of in CD8+ CAR-T cells, possibly via interleukin 10 (IL-10)-mediated immune suppression (33, 37). Similarly, the use of IL-18 secreting CAR-T cells enhances antitumor effects and generates a pro-inflammatory environment, while recruiting inflammatory cells without severe toxicity (37, 38).

The effects of IL-15 releasing TRUCKs offer promising therapeutic applications by favorizing a T stem cell memory -like phenotype, increased persistence, and antitumoral activity via BCL upregulation (33). Fifth generation CAR-T cells, in addition to second and third generations, rely on activation of JAK-STAT pathways via an additional truncated intracellular domain of cytokine receptors with a binding site for transcription factor STAT3 (39–41).

Though innovative and exciting, not all advancements guarantee better outcomes, as benefits gained in terms of cytotoxicity may be diminished by exhaustion through tonic signaling, and increased persistence mediated by interleukin secretion can lead to more severe cytokine mediated systemic toxicity. Ideally, the optimal design for CAR constructs should be validated by testing combinations of signaling domains, co-stimulatory regions in systematic head-to-head comparisons, though financial and economic constraints are limiting for this scale of trials.

### 1.1 CAR-T cell mechanism of action

**Figure 2:**
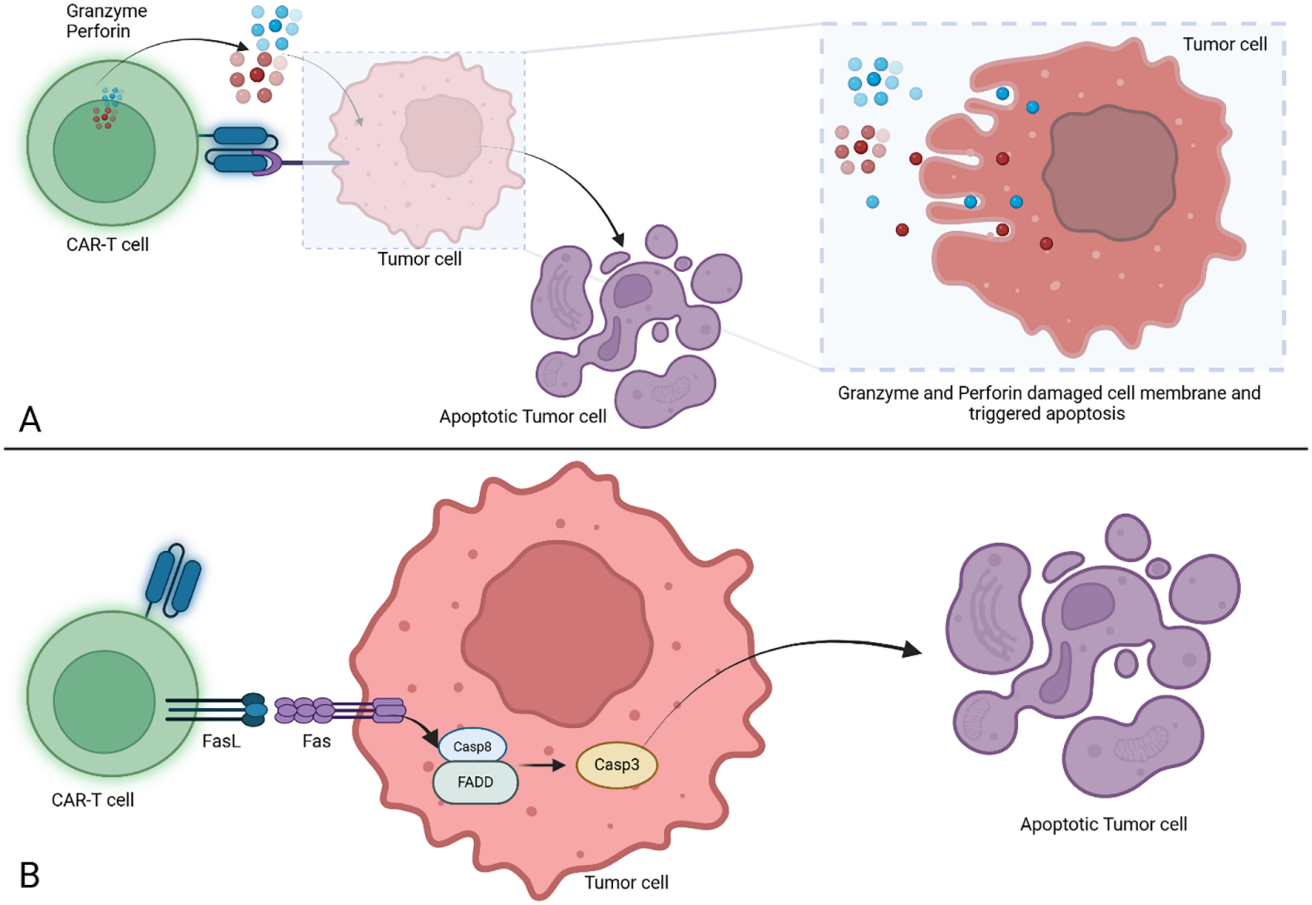
Cell death triggered by CAR-T cells. **(A)** Cell death induced by granzyme and perforin action. The CAR-T cells are releasing perforin and granzyme after binding the target, both are inducing membrane damage and trigger the apoptosis in tumor cells. **(B)** Cell death induced via FasL/Fas mechanism. The CAR-T cells trigger the FasL/Fas signaling which trigger the activation of Casp8 which will further initiate Casp3 cleavage leading to tumor cell apoptosis. CAR-T, Chimeric Antigen Receptor T cell; FasL, Fas Ligand; Fas, Fas receptor (known as CD95 or APO-1); Casp8, Caspase 8; FADD, Fas associated death domain; Casp3, Caspase 3.

Depending on generation, CAR-T cells fully or partially mirror the physiologic TCR mediated activation of T lymphocytes, with its 3 essential signals. Activation is initiated following recognition by the antigen recognition domain of its cognate antigen, constituting signal 1 and leading to immunoreceptor tyrosine-based activation motif (ITAM) phosphorylation in the CD3ζ domain. Signal 2 is provided by the costimulatory molecules, and optimal T cell functioning is achieved with the contribution of the 3^rd^ signal mediated by cytokines (42, 43). To exert their cytolytic effects, CAR-T cells employ two main pathways (**Figure 2**). The perforin and granzymes induce cell death by creating pores in tumor cell membranes which are used by the granzymes to enter the cytosol and trigger apoptotic death through caspase dependent and independent pathways. The second pathway makes use of FAS (CD95) ligand secreted by the T lymphocytes, which upon binding to its receptor on tumor cells, leads to the formation of a death-inducing signaling complex followed by cell death (43–45). Interestingly, FAS-FASL mediated cytolytic activity has been reported to be responsible for cytolytic activity against antigen-negative tumors as well, allowing for clearance of antigen-heterogenous tumors which might prove to be an avenue for overcoming mechanisms of resistance to treatment by antigen loss (46).

## 2 Available experimental models for CAR-T research

The Development of novel, effective CAR-T therapies can be a challenging task. For these therapies to be considered for clinical applications, first they must be thoroughly characterized. The purpose of these laborious processes is to predict as accurately as possible their behavior in the human body. Unfortunately, a model remains just that, and the multi-faceted characteristics of CAR-T cells require multiple *in vitro* and *in vivo* surrogates to be combined to achieve a comprehensive characterization.

### 2.1 Modeling the target

**Figure 3:**
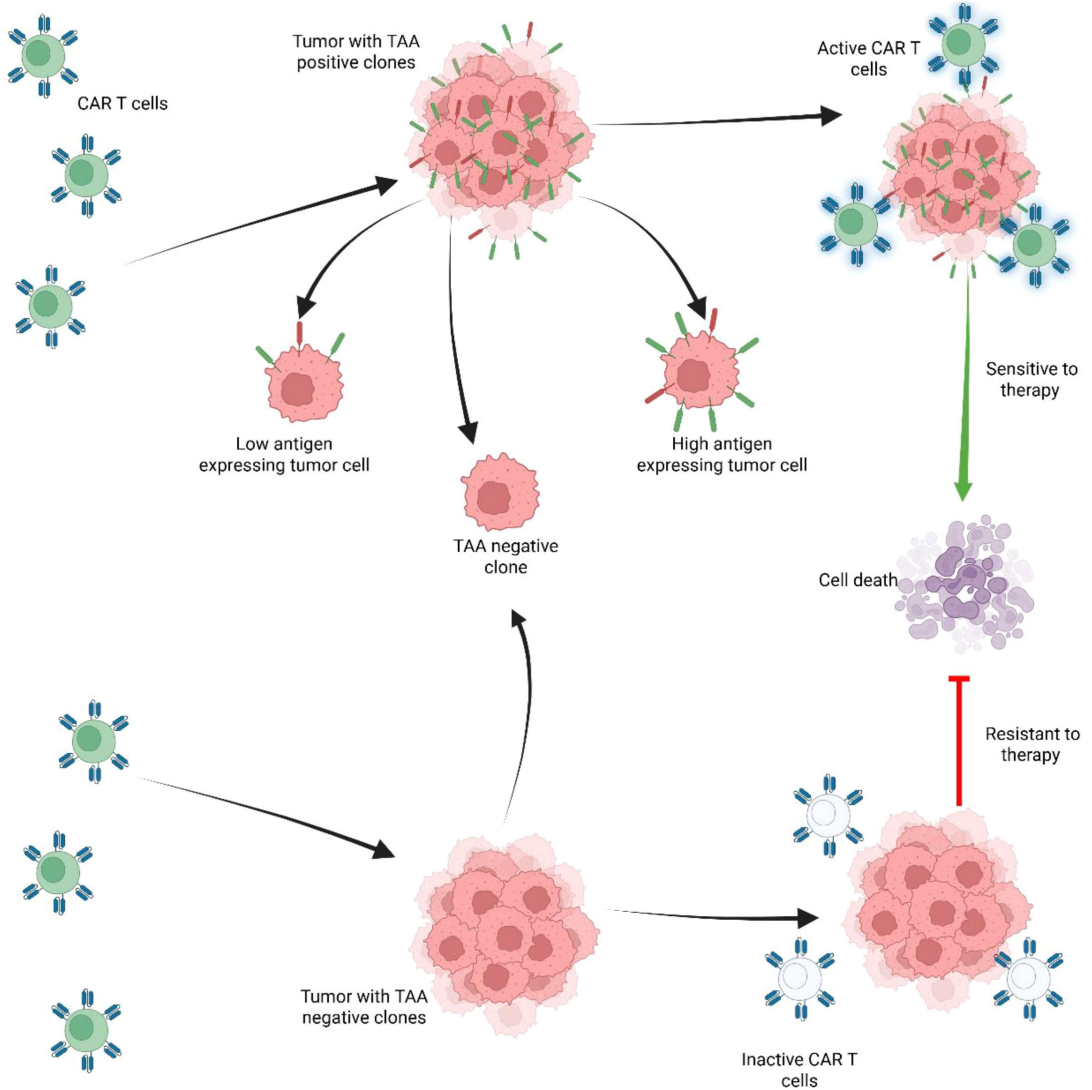
Expression of tumor associated antigens (TAA) regulates CAR-T cell persistence and response to therapy. Tumors with positive TAA clones are sensitive to therapy. However, tumors with poor response to therapy are associated with negative TAA and with other factors that may induce resistance, such as: T-cell exhaustion, senescence, or T-cell differentiation. In tumor with heterogenous tumor cells including population with high antigen expressing tumor cells and tumor cells that are TAA negative, the TAA negative clones are selected as they have poor response to CAR-T therapy and promote resistance to therapy, while TAA positive clones, are targeted and undergo cell death.

The cytotoxic potential of a novel CAR construct can be assessed *in vitro*, by using tumor-associated antigen (TAA) expressing cells, or with cell-free antigens. Plate or nanobead-bound recombinant antigens enable the isolation and evaluation of CAR-T cell activation in a strictly CAR dependent manner without the contribution of normally occurring costimulatory molecules and cell ligands. This also allows for easy adjustment of antigen density (47–49). Serving as a universal tool for CAR antigen binding is protein L, a protein of bacterial origin which indiscriminately binds to immunoglobulin light chain and scFvs, and can be used for CAR detection as well as for CAR mediated T cell activation (50, 51). Evaluation of cytotoxic activity against living cells is the mainstay of *in vitro* testing as this can provide a more complex view of CAR-T and tumor cell interaction, recapitulating costimulatory signaling, dynamics of cell killing and allows modulation of effector to target (E:T) ratios as well as thorough characterization of T lymphocytes. The most readily available and widely used experimental targets for CAR-T therapy are immortalized cells (cell lines). They are well characterized and easy to use, thus providing an important frame for various assays in CAR T development. Tumor cell lines can expand indefinitely and can be genetically engineered to express fluorescent reporter genes or knocked-out for certain genes to produce negative control targets (52–54). Additionally, target cells can be created by inducing expression of certain transgenic antigens. One such example is the acute B cell leukemia cell line NALM-6 which is often transduced to express tumor associated antigens (TAA) and to control antigen density (55) (**Figure 3**).

While immortalized cells offer a reliable model for research, they often harbor complex cytogenetic abnormalities and mutations, and their behavior may differ in certain aspects from *in vivo* counterparts (56). Primary tumor cultures offer an alternative *ex-vivo* approach which accurately replicates primary tumor biology. However, they bear certain limitations regarding cell purity, while issues of tumor tissue accessibility and limited low *ex vivo* culture potential limit the quantities of primary cells available for experiments. Additionally, repeated passages of primary cultures alter the cellular heterogeneity with preferential selection of subclones (57, 58).

### 2.2 Spheroid cultures (3D structures)

Bridging the gap towards a more realistic *in vitro* model of tumors are patient-derived organotypic spheroids (PDOS), a novel 3D *ex-vivo* model created from patient tumor tissues. This model is achieved by enzymatic and physical tumor tissue dissociation and cell separation, after which cells are resuspended in gel to reconstitute the 3D structure (59). Spheroids bear a high resemblance to the original tumor and preserve tumor multicellularity and its native niche. PDOS exhibits architectural heterogeneity, with superficial layers predominantly epithelial and an inner core of mesenchymal origin. The spheroids retain initial tumor cells with stem-like properties and are capable of engrafting in mice to produce tumor xenografts (60, 61). The utility of these 3D structures is more important in the development of solid-tumor targeted CAR-T therapies, as this system may replicate aspects of cell trafficking into tumors and temporospatial heterogeneity of CAR-T cells characteristics as well as the influence of local tumor niche on the adoptive cells. This provides a foundation for optimizing CAR-T cells. For example, Cho et al. (62) have shown that the size of breast cancer–derived organoids directly impact CAR-T cell intratumoral trafficking and cytotoxicity, with reduced cytotoxicity observed in the spheroid core (62). Additionally, supporting data for the ability of PDOS to model *in vivo* CAR-T efficacy comes from pioneering work by Logun et al., in which the *in vitro* cytotoxicity exhibited by CAR-T cells against patient-derived glioblastoma organoids mirrored CAR-T expansion and cytokine release patterns observed in the same patients during a phase I clinical trial (63).

Ideally, autologous CAR-T cells would avoid any alloreactivity that could confound results in PDOS settings. However, using T cells autologous to the patient from whom the PDOS are derived is challenging and uncommon. Allogeneic CAR-T cells used in these models must account for TCR-mediated non-specific cytotoxicity, which can be controlled by including appropriate experimental controls, such as non-specific CAR-T cells or non-transduced T cells from the same donor (64). Alternatively, TCR-mediated cytotoxicity can be mitigated by using TCR-knockout CAR-T cells while preserving CAR-specific activation (64).

### 2.3 *In vivo* models

Adequate *in vivo* models are essential for bridging *in vitro* research to clinical applications, to mitigate potential adverse effects such as cytokine release syndrome (CRS) and immune effector cell associated neurotoxicity as well as to validate antitumor effects taking into consideration tumor architecture, heterogeneity and influence of tumor microenvironment. Additionally, important data such as tumor infiltration, dynamics of CAR-T cells persistence in the host organism can be obtained using animal models (48, 65).

#### 2.3.1 Immune-compromised models – xenograft models

Engraftment of human tumors in immune-compromised mice is the core principle of xenograft models, the most used *in vivo* model for CAR-T cell research. Human tumors can be obtained in mice by inoculation with immortalized human cell lines or primary tumors for creating patient-derived xenografts (PDX) (66). Inoculation can be done intravenously, to replicate metastases, subcutaneously, for localized tumors, providing easy access, or orthotopically, for more anatomically realistic models. Mice used in for xenograft models are all deficient in adaptive immunity and including T lymphocytes, thus unable to mount a host versus graft reaction, and rejection of foreign tissues (67). While athymic nude (nu/nu) mice lack T lymphocytes, severe combined immunodeficient (SCID) mice lack both B and T cells, and other types, such as non-obese SCID, Rag2-Knockout and NSG Mice (NOD-SCID IL2Rγnull) have increasingly more profound immune suppression, making them more suitable for the engraftment of patient-derived tumors (68, 69). Retaining part of the myeloid-derived immune cells makes it possible to evaluate CAR-T therapies considering the influence of myeloid-derived suppressor cells (MDSC) such as dendritic cells and macrophages on tumors and adoptive cells. This is only valid to some extent, and it is also noteworthy that these MDSCs may not be fully competent, thus not being able to fully mirror the properties of human MDSCs (70).

Though mice are the most common *in vivo* models, they are largely unfit for the evaluation of adverse effects of adoptive cell therapy, especially regarding CRS, a severe and potentially lethal complication caused by large-scale immune cell activation (71). Thus, non-human primates, though expensive and less accessible, are used as immunocompetent models for more reliable recapitulation of CAR-T cell-associated toxicities. They also serve as models for novel approaches, such as *in vivo* CAR-T cell generation, which require competent host T cells and higher resemblance to humans (65, 72). A cheaper xenograft alternative to mice, with higher throughput, is the zebrafish embryonic xenograft, which lacks a functional adaptive immune system while in this stage of development. These models have important limitations related to their short duration for evaluation, and due to important differences between human and zebrafish signaling pathways. Nonetheless, they have been successfully used to assess CAR-T mediated antitumor effects *in vivo* and offer the advantages of requiring small tumor samples, a low number of CAR-T cells, and allow high-resolution live imaging of effector: target (E:T) interactions (73, 74).

Importantly, significant differences have been observed in response to CAR-T therapies between *in vivo* models and human trials. arise due to omission of TME. This discrepancy can be attributed to the exclusion of the influence of the tumor microenvironment (TME) on tumor survival, progression, and resistance development in *in vivo* immunodeficient models as it happens in immune-deficient mouse models (75). The complexity of TME and tumor tumor-associated macrophages (TAM) interaction with CAR-T cells is gaining increasingly more attention due to their important immune suppressive effects, limiting CAR-T efficacy. Alternatively, the potential of cytokine-producing CAR-T cells to recruit inflammatory cells and modulate an antitumoral microenvironment makes comprehensive TME-tumor models essential in preclinical research (36, 76). The prerequisite for this is the presence of a functional, adaptive, and innate immune system. However, this implies the ability to mount a graft-versus-graft effect.

### 2.4 Modeling the target and microenvironment

#### 2.4.1 Immune competent - humanized

Humanized mice (HM) are obtained via reconstitution of the human immune system in immune-deficient mice and are achieved through the engraftment of human CD34+ hematopoietic stem and precursor cells (HSC) in mice. This allows the engraftment of a human tumor in immune competent mice and a more accurate portrayal of tumor-TME interaction. Depending on the origin, engraftment of HSCs from bone marrow, peripheral blood, cord blood, and bone-liver-marrow cells differ in terms of complications and duration of immune reconstitution, and achieve slightly different mature populations (77). Following differentiation, myeloid and lymphoid cells can interact with and infiltrate tumors, recapitulating the TME. Unfortunately, HM are expensive, difficult to obtain and are accompanied by significant limitations constraints. These include the limited availability of human stem cells, the toxicity of chemotherapy or irradiation conditioning, and the risk of engraftment failure. Additionally, they may be complicated by xenogeneic graft versus host disease (GVHD), especially as mice age (77–80).

#### 2.4.2 Immune competent – syngeneic

Tumor-bearing immune competent mice fully reconstitute the complex TME and are easily obtained by inoculation of mice with murine tumors of genetically identical background (81). Additionally, genetically engineered mouse models (GEMMs) such as the Vk*MYC or the Tyr(CreER, BrafCA, Ptenf/f) develop tumors spontaneous spontaneously or under certain stimuli, recapitulating oncogenesis with high fidelity, albeit tumors produced this way present with reduced immunogenicity (82–84). By being the closest to nature *in vivo* model, in addition to TME models, syngeneic mice are indispensable for modeling and understanding lymphodepletion prior to CAR-T cell administration and for assessment of on-target-off-tumor toxicity and CRS (85, 86). The limitations of syngeneic mouse models stem from the compromise that both tumors and CAR-T cells are of murine origin. This implies notable differences regarding immune cells and cytokine function when compared to humans (87, 88). Antigen expression varies between the two species, and certain epitopes have different immunogenicity and thus, targets are not always translatable between mice and humans (84–89). An alternative approach in such instances is the use of transgenic mice, which can be genetically engineered to express human antigens in an immunocompetent all-murine setting (74). The main drawback of syngeneic models remains the difficulty of obtaining murine CAR-T cells, as current protocols for murine T culture and expansion have low yields, which is only aggravated by lower cytotoxicity and shorter persistence (85–90).

### 2.5 Modeling the effector

In addition to target tumor models used in CAR-T research, models for effector cells offer provide a reliable setting framework for the assessment of novel CAR constructs. Acute T cell leukemia cell line, Jurkat is particularly useful for this purpose as it lacks TCR α and β chain, which can prove effective to avoid possible Graft versus Host Disease (GVHD) toxicities and TCR-mediated T cell activation (91, 92). CAR-Jurkat cells have been successfully used to show anti-tumor effects of novel CAR constructs and have the advantage of being high-throughput (93, 94).

The advantages of using an established cell line for CAR characterization stem from the sturdiness of cells, allowing for transduction with multiple reporter genes as well as for selection and expansion of CAR-transduced cells to obtain a homogenous cell population (95, 96). While this model is far from the reality in the way that it does not recapitulate all the various T cell subtypes obtained from patient peripheral blood mononuclear cells (PBMC), as well as missing the inter-patient CD4+/CD8+ variability, it allows characterization of CAR constructs while reducing background noise through isolation of CAR mediated T cell activation (97).

It is increasingly evident that model selection dramatically influences preclinical CAR-T outcomes. Immortalized cell lines are high-throughput models though with no heterogeneity; primary tumor cultures are more clinically relevant but are subject to clonal drifting; PDOS exhibit spatial heterogeneity by they lack systemic features, like CAR-T persistence and immune system interactions whereas mouse models either humanized or syngeneic are expensive, time consuming and are prone to GVHD or require murine CAR constructs respectively. As such, integrated experimental strategies are crucial and tiered model pipelines are the future for CAR-T therapies to ensure translational relevance and align preclinical data with clinical results (91, 92, 97).

## 3 Methods for identifying novel CAR-T target

Despite the remarkable initial success of CD19 and B cell maturation antigen (BCMA)-targeting CAR-T cells in the treatment of B-cell derived malignancies, long-term follow-up studies reveal that not all patients achieve durable responses, partially due to downregulation or loss of target antigen (98, 99). The increasing interest in CAR T cell therapies in oncology promotes extensive investigation for new surface proteins that could be targeted by adoptive cell immunotherapies. However, finding an appropriate surface antigen carries various challenges. An ideal target for CAR-T-mediated immunotherapy should exhibit high, exclusive, and uniform expression on cancer cells, including cancer stem cells. To provide low off-tumor activity and avoid life-threatening toxicity, the target shall not be present in vital tissues, nor be expressed on cells responsible for normal hematopoiesis (hematopoietic stem/progenitor cells (HSPCs)) (100). Furthermore, a successful CAR target must be associated with high stability and sustainability. This could be accomplished by identifying antigens that play essential roles in cancer biology and survival. It is noteworthy however that none of the proteins targeted by the currently approved CAR-T therapies fit all of these requirements, particularly since tumor-specific antigens are rare.

Despite these challenges, an increasing interest in finding novel targets for CAR-based immunotherapy has been observed over the years, both for new indications (e.g., solid tumors) (101) and for relapsed/refractory (r/r) hematological malignancies in which previous CAR-T treatment rendered ineffective due to antigen escape (99, 102). Indeed, since seminal case report studies with CD19 CAR-T cells were published in the early 2010s (7, 103–105), the number of clinical trials targeting surface proteins expanded exponentially. Thus, as of the end of 2024 there are 169 ongoing, and nearly a thousand clinical trials still looking for participants (https://clinicaltrials.gov/). Selecting known cancer biomarkers or surface proteins targeted by already existing clinically approved immunotherapies, especially monoclonal antibodies (mAbs), was one of the earliest strategies to create new CAR-T treatments for pre-clinical evaluation. Examples of such antigens include known surface bio-/prognostic markers such as mesothelin (106, 107), PSMA (108), GPRC5D (109, 110), or previously known immunotherapeutic targets: CD20 (111), HER2 (ERBB2) (112), and EGFR (113), targeted clinically with rituximab, trastuzumab, and cetuximab mAbs, respectively. Importantly, as the mechanism of action of mAbs and CAR-T cells differs greatly, targeting the same antigens through various effector immune cells may provide different and unexpected toxicity profiles. Indeed, infusion of anti-HER2 CAR-T cells resulted in multiorgan failure in a patient with metastatic colon cancer due to rapid cytokine release following target recognition on normal lung cells (114). Simultaneously, anti-HER2 mAbs (e.g., trastuzumab, pertuzumab, margetuximab) are safely used for patients with HER2-positive breast cancer alone or in combination with chemotherapy (115). Similar observations were made for other antigens, including CD38 and PD-L1. Despite the successful targeting of these proteins with respective mAbs, daratumumab (CD38) (116) or atezolizumab (PD-L1) (117) in clinical practice, case report studies demonstrated life-threatening toxicities in patients infused with anti-CD38 (118) or anti-PD-L1 (119) CAR-T cells. Ultimately, this data underscores that target identification for CAR-T therapy must be performed with caution and rigorous pre-clinical evaluation, employing malignant and normal cells. Therefore, in this chapter, we will summarize unbiased approaches to actively searching for cancer-associated and cancer-specific proteins, which have led to the development of new CARs.

Given the considerable advances achieved in studying the transcriptome of human malignancies and the increasing accessibility of high-throughput methods such as RNA sequencing (RNA-seq), the search for novel cancer biomarkers has long relied on these tools. Importantly, since RNA-seq evaluates the level of all transcripts in the cell, established tools for the annotation of surface protein-coding genes are crucial for appropriate CAR target identification (120). Furthermore, the employment of transcriptomic data for immunotherapy target selection suffers inaccuracy due to a complex correlation between transcript expression and protein level in cells, which is owing in part to varying transcript isoforms and translation efficiency (121). Therefore, to minimize the inaccuracy of this approach, integrated transcriptome-proteome analyses of normal and cancer cells have been proposed (122). Indeed, Perna et al. (123) presented an elegant pipeline for CAR target selection, integrating transcriptomic and proteomic data generated from acute myeloid leukemia (AML) cell lines/patient samples, a comprehensive literature search of already published CARs, and available databases of protein levels in normal tissues. The rigorous algorithm served to identify more than 20 potential CAR targets. Expression of these proteins was then evaluated by flow cytometry in primary AML samples, normal bone marrow, and resting/activating T cells to exclude the possibility of fratricide killing mediated by CAR-T cells. Accordingly, four molecules, ADGRE2, CCR1, CD70, and LILRB2 represented the best profile of expression, fulfilling most of the criteria for the desirable CAR candidate described at the beginning of this chapter. In a subsequent study, the authors verified the expression of selected targets in r/r AML patients and successfully designed CAR-T cells targeting ADGRE2 in combination with CLEC12A (124). Importantly, several other targets were discovered by the combined transcriptomic/proteomic approach, such as CCR10 (125), ILT3 (LILRB4) (126), and endothelin receptor B (127), all in multiple myeloma cells. Accordingly, for CCR10 and LILRB4, antigen-specific CAR-T cells were developed and proved effective in pre-clinical studies (125, 128).

Nevertheless, owing to the extensive technological progress that has been made in studying cell surfaceome, several recent studies relied entirely on proteomic data in the search for new CAR candidates (129–131). comprehensive analyses employing mass spectrometry (MS) platforms are currently well-recognized in the CAR-T field and are superior to conventional flow cytometry and mass cytometry approaches as they are not restricted to the necessity of using previously generated antibodies. Surfaceome profiling is a multistep procedure aimed at the specific enrichment of surface proteins, which are then analyzed with liquid chromatography-tandem mass spectrometry (LC-MS/MS). The capture of surface proteins is achieved through various techniques, with chemical-based tagging being the most common (132). This includes approaches based on biotinylation, metabolic labeling, or cell-surface capture by glycan oxidation. A comprehensive and elegant summary of these and other MS-based techniques for immunotherapy target identification is available elsewhere (133).

Cell surface enrichment of malignant cells, followed by MS, contributed to the discovery of new immunotherapy targets, such as CD72 in B-cell acute lymphoblastic leukemia (129) or SEMA4A in multiple myeloma (131, 134). In addition, Mandal et al. (130) presented a specific form of proteomic approach aimed at identifying tumor-specific proteins, focusing on structural differences in surface antigens of cancer and normal cells. Interestingly, the authors combined cross-linking mass spectrometry (XL–MS) with the cell surface capture method, thus yielding enriched surface N-linked glycoproteins in their native conformation. This led to identifying AML-specific, activated integrin β2, and generating a novel CAR-T cell therapy, thoroughly tested in preclinical studies. Of note, one of the challenges of this structural proteomics technique and other MS-based approaches for studying cancer cell surfaceome lies in the high sample input required. As a result, the majority of proteomic studies mentioned in this review used human-immortalized malignant cell lines. This approach, however, does not recapitulate cancer heterogeneity observed in patients nor capture all attractive antigens, which may be absent on established cell lines. Noteworthy, Marhelava et al. described an optimized method for cell surface biotinylation, subsequent MS, and surface protein detection on xenograft cells generated from B-cell acute lymphoblastic leukemia patients (135).

Moreover, an innovative approach has been recently developed to guide CAR-T cells to neuroblastoma cells (136). In the seminal paper, the authors screened the immunopeptidome of patient-derived xenografts and found that PHOX2B oncogene-derived peptides presented in specific MHC class I molecules (HLA) were particularly enriched in tumors. Interestingly, as selected peptides were not immunogenic and peptide-specific TCRs did not exert high affinity, peptide-centric CARs were designed. The selection of scFvs binding PHOX2B peptide-MHC (pMHC) complexes was performed, which resulted in identifying one tumor-specific binder. Importantly, PHOX2B-peptide-centric CAR-T cells showed impressive tumor-killing potential in pre-clinical neuroblastoma xenograft models with different HLA allotypes. This study highlights that integrated transcriptomic, epigenomic, and immunopeptidomic dataset analyses hold promise in searching for cancer-specific proteins that could be targeted with CAR-T cells. More clinically relevant data are needed to verify the safety and efficacy of this method.

Importantly, all above-mentioned techniques study the whole tumor population, thus failing to address tumor heterogeneity. The current advancement in single-cell analysis technologies overcomes issues and provides a helpful tool to profile the tumor at a single-cell resolution. This is particularly important as bulk tumor analysis for CAR target identification may overlook rare, though clinically important cell types, such as cancer stem cells or therapy-resistant clones. Noteworthy, cover single-cell transcriptomics (scRNA-seq), with various platforms available, such as 10X Genomics. Indeed, by using scRNA-seq datasets, Gottschlich et al. identified CSF1R CD86 as viable CAR targets for AML (137). It is important to note that, given the complex correlation between transcript expression and protein level, single-cell proteomics are arguably more useful for developing CAR targets than single-cell transcriptomics. In fact, single-cell proteomic techniques such as CITE-seq (cellular indexing of transcriptomes and epitopes by sequencing) (138) or Cellenion’s platforms have been developed and could be exploited to revolutionize CAR target development (139).

Nonetheless, despite impressive numbers of novel techniques for CAR target selection and novel CAR-T therapies being tested as single or multi-targeting CARs (dual, tandem, mixed, etc.), thus addressing tumor heterogeneity, other hurdles related to CAR-T treatment persist. These challenges are particularly frequent in solid tumors and are linked to limited CAR-T cells trafficking and persistence in the tumor microenvironment, as explained in detail in the following chapter.

## 4 Challenges for CAR-T cells in solid malignancies

### 4.1 Limited efficacy in clinical response

CAR-T cell therapies have made a name for themselves and first gained approval for use in hematologic malignancies though initial studies did not specifically aim a narrow spectrum of malignancies. In fact, some of the earliest targets for CAR-T research were solid tumors such as ovarian cancer, colorectal carcinoma and renal cancers, however several core differences between solid and hematologic malignancies have favored the latter for clinical applications of CAR-T cells which gained approval for clinical practice whereas, to date, no CAR-T therapies are FDA approved for solid tumors (140, 141). Unfortunately, despite exciting results *in vitro*, early phase clinical trials for solid cancers showed little to no response. Consequently, a significant amount of research is currently being undertaken to elucidate the underlying causes of this phenomenon. and to date much research is going into decrypting the reasons for this matter. For instance, a phase I trial of CAR-T cells targeting the α folate receptor in 8 patients with metastatic ovarian cancer, and another targeting the tumor-associated glycoprotein 72 (TAG-72) for metastatic colon cancer showed no clinical response. However, in the latter trial, the longest living out of the 25 patients was the patient with the greatest most significant lymphocyte expansion and had detectable circulating CAR-T cells at 48 weeks and whereas in all other patients they were not detectable after 14 weeks, thus pointing to potential benefits of CAR-T cells in solid tumors if their activity can be preserved. Despite the low or absent objective clinical responses, these trials did much to show that adoptive immune therapies in solid tumors are a category of their own when it comes to CAR-T cell efficacy, or lack thereof. Low CAR-T persistence, reduced intratumor trafficking and the occurrence of inhibitory factors to CARs all rapidly emerged as challenges which would require various strategies to be overcome (16, 142).

### 4.2 CAR-T cell expansion and persistence

CAR-T cell expansion as an early activity indicator, followed by persistence are clearly associated with favorable responses in hematologic malignancies (143). Stemming from the use of murine-derived antibodies and their inherent immunogenicity, the occurrence of human anti-chimeric antibodies (HACA) hindering T cell expansion, has been reported in several clinical trials, with over half of the patients developing CAR-directed antibodies in clinical trials targeting TAG-72 (142). In another clinical trial, with similar incidence of HACA, investigators showed that antibodies arising to CAR-T targeting carbonic anhydrase IX (CAIX) have inhibitory capacities and reduce CAR-T functionality and persistence (144). Though not specific to solid tumors, this phenomenon seems to be reported less frequently in hematologic malignancies. This might have to do with the prior treatments that patients with lymphoma and leukemias often undergo prior to CAR-T therapies and are therefore often more lymphopenic than patients with solid tumors. Lymphodepletion with Cyclophosphamide (CP) and Fludarabine (FLU) has become an integral part of CAR-T therapies as it led to remarkable benefits across trials in both types of cancers, allowing for achievement of 72% overall response rate (ORR) and 50% complete remission (CR) in relapsed refractory Non-Hodgkin Lymphomas (NHL) treated with CD19 targeted CAR-T therapies, with enhanced T cell expansion as well as reducing immune responses to therapy (145). The efficacy of conditioning judged by the degree of lymphopenia at the time of adoptive cells infusion appears to be good predictor for T cell engraftment, as absolute lymphocyte numbers are inversely correlated with CAR-T expansion (146). For instance, a phase I trial using conditioning with either CP+Oxaliplatine or CP+FLU showed more profound lymphodepletion with the latter regimen, which correlated with higher peak CAR-T expansion as well as lower immune response to CAR sequence (147). Likewise, in two phase I solid tumor trials targeting CEACAM5+ cancers and metastatic castration resistant prostate cancer, prior conditioning with FLU and CP or CP alone led to improved T lymphocyte expansion and activation in patients with more intense conditioning, however, both trials reported serious adverse effects of acute respiratory toxicity and CP dose-related cystitis respectively (148, 149). Perhaps due to the rather intact adaptive immunity of patients with solid tumors, the maximal benefits of lymphodepletion cannot currently be achieved due to dose-limiting toxicities. Indeed, there may yet be benefits to be achieved with alternative conditioning regimens.

Persistence of CAR-T cells after infusion is a particularly challenging aspect in solid tumors. With circulating tumor cells readily available, hematologic malignancies are naturally more accessible targets, and the hypothesis is that persistent antigen exposure is what entertains enables superior CAR-T cell expansion and persistence in these patients (150). Generally, the kinetics of infused cells follow pattern of expansion at 7-10 days, followed by a gradual decrease to undetectable levels at approximately 6 weeks (91, 151). A phase I/II trial for HER-2 positive sarcoma included 19 patients, maximum CAR-T levels were observed at 3 hours following infusion and persisted for 6 weeks, however, no expansion was observed. Despite this, tumor samples from two patients, obtained following treatment, both showed CAR-T infiltration (152). In another phase I trial targeting EFGRvIII in recurrent glioblastoma, including 10 patients, the peak expansion occurred within 3-10 days and was followed by a rapid decline after the 14^th^ day. Seven of the 10 patients underwent surgical tumor resection at different time points, which allowed for assessment of tumor CAR-T infiltration. Interestingly, tumor infiltration seems to be higher at the earlier time points, suggesting that there is no late CAR-T localization in the tumors in this case (151). While persistence has become an indicator of promise and efficacy for CAR-T, it does not always seem to be the case. When evaluating GD.2 targeting CAR-T including a constitutively active chimeric IL-7 receptor in high grade pediatric tumors in a phase I trial, patients experienced improvement in neurologic deficits and 29% of 11 patients achieved objective partial response, however response to treatment did not show any correlation with expansion in peripheral blood and while circulating CAR-T cells declined within 4 weeks, they were present in tumors up to 3 months post infusion (153). These observations imply that due to tumor-localized antigens, peripheral CAR-T cell persistence in patients with solid tumors is a surrogate and might not capture the dynamics within tumors and lymphoid structures.

### 4.3 CAR-T cells intra tumoral trafficking

A very relevant depiction of the dual nature of prerequisites for CAR-T cell therapy efficacy in solid tumors comes from the biology of checkpoint inhibitors and mechanisms of resistance to treatment.

According to work done by Dangaj et al. characterizing the immune reactivity of tumors, the efficacy of checkpoint inhibitors is dependent on tumor infiltration by cytotoxic T cells. The key players in these events are the chemokines CCL5 and CXCL9 secreted by tumor cells and local myeloid cells respectively. Overexpressing tumors are immunoreactive and are associated with improved outcomes and response to checkpoint inhibitors, whereas downregulation of chemokine expression via DNA methylation leads to loss of infiltrating lymphocytes (154). In a complementary manner, murine studies of pancreatic ductal adenocarcinoma showed that residing cancer-associated fibroblasts (CAF) as well as FAP (fibroblast activation protein) positive stromal cells reduce the efficacy of checkpoint inhibitors by suppressing the cytotoxic activity of locally present cancer specific effector T cells. This inhibition is mediated by secretion of CXCL12 binding to CXCL12 receptor on tumor cell but antitumoral effects of checkpoint inhibitors can be restored via depletion of CAF or inhibition of CXCL12 (155).

As checkpoint inhibitors mechanism of action relies on endogenous cytotoxic T cells, it becomes evident that the intra tumoral presence of reactive T cells and their actual anti-tumor effects are two distinct prerequisites for CAR-T cell therapy success. The barriers preventing these goals for CAR-T cells are described as reduced intra tumoral trafficking and local immune suppression under the influence of the local TME.

Encompassing the stark differences in persistence and trafficking between hematological and solid malignancies is an interesting phase I clinical trial which used the same ROR1 targeting CAR-T cells in patients with ROR1 positive chronic lymphocytic leukemia (CLL), breast cancer and non-small cell lung cancer. This particular setting allows for a fairer comparison between the two different entities. As expected, expansion was greatest in CLL patients with the highest peak (over 95% of CD8+ cells) in the patient with the highest proportion of circulating tumor lymphocytes perhaps due to increased antigen exposure, whereas peak levels in patients with solid tumors were much lower, and 4 out of 18 patients had peak CAR-T levels < 3% of circulating CD8+ cells. This translated into trafficking, with only 2 out of 7 solid tumor samples showing detectable CAR-T levels and this was in the patients with high expansion peaks. Two out of 3 patients with CLL achieved a partial response, whereas, disappointingly, only one out of 18 patients with solid tumors achieved a transient partial response (147). The underwhelming levels of tumor infiltration seem to be improving with the use of novel generations of CAR-T cells (152). For instance, a clinical trial using PSMA TGFβ dominant negative armored CAR-T cells showed better tumor trafficking, detectable in 7 out of 9 biopsies performed at day 10 following infusion. The CAR-T levels measured by qPCR as copies/ng of genomic DNA were 1 log lower than in peripheral blood in most patients, whereas one patient had 17 times higher CAR-T levels in tumor than in blood, however despite approximately 30% of patients showing a reduction in PSA, no radiological response was documented (156). Though CAR DNA can be found in increasingly more samples, the small size of patient cohorts is insufficient to make correlations with clinical response, which is made more difficult by the very low number of responders. Multiple studies have shown both in murine models as well as human trials that local administration of CAR-T cells enhance trafficking and antitumoral effects, although it is still unclear what appropriate tumor infiltration is and will probably vary depending on tumor and particular CAR construct.

Inherent to the heterogenous and tridimensional nature of solid tumors, infiltration of the neoplastic fortresses is a monumental task for transgenic lymphocytes. The first challenge encountered by CAR-T cells is the lack of physiological stimuli to guide lymphocytes to inflammation sites. Selective extravasation of lymphocytes from circulating blood into tissues is dependent on endothelial upregulation of integrins and selectins and is also supported by expression of costimulatory molecules. In tumor vessels, angiogenic factors VEGF, bFGF mediate a reduction in expression of integrins ICAM-1/2, VCAM-1, and E selectin leading to the so called anergy manifested as reduced lymphocyte-endothelium interaction and immune tolerance. Additionally, tumors can induce endothelial cells to secrete Fas-ligand which further reduces lymphocyte infiltration by inducing apoptosis in the adhering cells (157). Secondly, a physical barrier of dense tumor stroma and extracellular matrix produced by fibroblasts isolate tumors from the immune cells (158).

It stands to reason that antitumoral effects would be directly correlated with the number of CAR-T cells located inside the tumors. However, assessing effector cell trafficking to tumor sites proves to be rather difficult and currently available data on CAR-T trafficking in clinical trials is very scarce. Very few studies include systematic biopsies while others assessed effector cell infiltration on biopsies obtained from patients undergoing surgery mostly for palliative reasons.

Accurate assays are critical to understanding and optimizing CAR-T therapies in solid tumors. Most accurate for this purpose are tumor biopsies which can be processed by immune histochemistry (IHC), flow cytometry of dissociated tissue or by more sensitive qPCR (159–161). The risks associated with repeated surgical sampling, potential infections and discomfort make it an unreasonable approach for routine practice and even for dynamic CAR-T monitoring within clinical trials.

Non-invasive assays would be much more practical for this purpose; however, they assays are not as sensitive as tumor biopsies. For instance, one clinical trial which used both biopsy and imaging found intra tumoral trafficking in one out of the three tumor samples, whereas 111-Indium based assays failed to show any tumor infiltration (142). As opposed to diagnostic applications of PET imaging, where its sensitivity is critical for evaluating residual disease, the purpose in CAR-T therapies would be to assess sufficient or relevant tumor infiltration, thus different expectations might be applicable in this case.

Various assays are available for *in vitro* and *in vivo* models, however, very few translate to human applications. Bioluminescence assays are commonly used in mice and make use of Luciferase transduced CAR-T cells able to emit light upon metabolization of substrate. Humans, however, are too large for the lymphocyte emitted light to traverse tissues. Two-photon microscopy, one of the highest resolution assays used in research is also not translatable to humans (51). Positron emission tomography (PET) based imaging is an alternative non-invasive assay which is reported to retain sensitivity for as few as 10000 CAR-T cells, which has been used in several clinical trials (162, 163). For this, CAR-T cells can be labeled prior to infusion and tracked after infusion for as long as they remain radioactive. This has no apparent deleterious effect on cell activation or viability; however the radiotracer is diluted with each cell division and though radioisotopes with long half-lives such as 89Zirconium-oxine can be used, the trafficking window is about 8 days (163, 164). Alternatively, CAR-T cells can be traced at any time point with the transduction of reporter genes which metabolize and accumulate radioactive substrate. Two such examples are reporter herpes simplex virus type 1 thymidine kinase (HSV1-TK) and probe 9-(4-(18F)fluoro-3-(hydroxymethyl)butyl)guanine (18F-FHBG) or Escherichia coli dihydrofolate reductase enzyme (eDHFR) reporter with (18F)-TMP fluorine-18 probe which have been validated for tracking CAR-T cells into tissues and confirmed by IHC (51, 165). The caveat of this approach is that CAR-T cells require an additional transduction prior to infusion, and that it cannot be applied to CAR-T cell therapies already in trials. Additionally, signal intensity in tumors seems to be influenced by local vascularization which may be low in poorly irrigated tumors. This is further complicated by the reported nonspecific tracer uptake in tissues leading to background signal (163). Inducible T-cell COStimulator (ICOS) targeting tracers directly bind activated T cells, thus obviating the need for prior CAR-T cell manipulation and allow tracking of CAR-T cells distribution though they will also show non-transduced T cells (166).

### 4.4 Tumor immunosuppressive microenvironment

Poor responses to therapy even in patients with detectable tumor infiltration confirm that the mere presence of CAR-T cells is not sufficient to produce adequate anti-tumor effects. Inactivation of CAR-T cells with the occurrence of exhausted phenotype is the result of both intrinsic and extrinsic factors. Excessive signaling attributable to the CAR structure itself has been shown to lead to exhaustion through tonic signaling, with 4-1BB CAR seemingly less affected by this phenomenon (167). Additionally, extrinsic signaling and immune suppression can induce T cell exhaustion, for instance through PD1/PD-L1 signaling (168).

As has been shown in the case of checkpoint blockade inhibitors, tumor microenvironment plays an important part in suppressing immunity towards tumors. Multiple cell types mediate the immune suppressive local microenvironment, with cancer associated fibroblasts (CAF), lymphocytes, endothelial cells, macrophages, and myeloid-derived suppressor cells (MDSC) altering cell phenotypes and functions to create a protective niche for cancer cells. MDSC seem to be especially important as they appear to expand in response to robust CAR-T cell expansion, protecting tumors (146, 156).

The immunosuppressive TME is characterized by the presence of various immunosuppressive cells such as regulatory T cells (Treg cells), myeloid-derived suppressor cells (MDSC) and tumor-associated macrophages, as well as the upregulated expression of immunosuppressive molecules such as programmed cell death protein 1 (PD-1) and programmed death-ligand 1 (PD-L1), making this environment an important barrier for an effective antitumor immune response (276–278). Treg cells are an immunosuppressive subset of CD4+ T cells characterized by the expression of the master transcription factor forkhead box protein P3 (FOXP3)+ and CD25 (the interleukin-2 (IL-2) receptor (chain)) (279). Treg cells were originally identified in 1995 by Sakaguchi et al. as CD4+CD25+ T cells that suppress an excessive immune response to various antigens but also contribute to tumor progression by inhibiting antitumor immunity (280). Treg cells are frequently detected in inflamed tumors, where they suppress various types of effector lymphocytes, including CD4+ T helper cells (TH) and CD8+ cytotoxic T lymphocytes and CD8+ cytotoxic T lymphocytes (CTLs) (281). In addition, tumor infiltration of Treg cells and the high number of Treg cells in the TME are associated with poor prognosis in various cancers (279, 282).

Interestingly, in a clinical trial including patients with recurrent glioblastoma, early tumor CAR-T trafficking was accompanied by polyclonal lymphocyte infiltrates, however these reactive lymphocytes show a Treg (regulatory) phenotype along with high concentration of immunosuppressor molecules (151). On the other hand, a trial using 4^th^ generation GD.2 targeting CAR-T cells for high grade pediatric tumors showed that incorporating a constitutively active IL-7 receptor leading to improved tumor cell killing was associated with higher level of tumor-specific polyfunctional cells (153). Similarly, another study in patients with recurrent high-grade glioma showed an increased survival associated with elevated pretreatment intra tumoral CD3 levels (146). This is to show that local immune cells are crucial allies which can play a dual role both pro and antitumoral and that treatment efficacy may depend on which way they can be swayed. One remarkable example of immune cells which can change allegiances is the tumor associated macrophage which can take a proinflammatory and antitumoral M1-like phenotype or a myelosuppressive M2-like phenotype which prevents T lymphocyte mediated cytotoxicity by secreting PD-L1 and CTLA4-lingands and is associated with poor prognosis (169, 170).

### 4.5 Tumor antigen heterogeneity

Unlike hematologic malignancies where lineage specific antigens are universally and consistently expressed, solid tumors lack highly specific targets (171). Instead, they are TAA, defined by overexpression, although these antigens are also shared by other normal tissues of epithelial origin. In addition to the lack of specificity, TAA exhibits important heterogeneity in expression levels between different patients but also within different regions of the same tumor and temporal heterogeneity with tumors changing histology over time. This is explained by selection of subclones and results in distinct tumor cell populations with varying levels of antigen expression (172–174). Tumor cells evasion of cytotoxicity through antigen expression downregulation and selection of TAA negative clones, termed antigen escape is one of the main mechanisms of resistance to CAR-T cell therapies.

For instance, the early recurring tumors in mice bearing peritoneal ovarian cancer showed reduced TAA expression and correlated with reduced CAR-T persistence and in patients with recurrent glioblastoma, five out of seven biopsies evaluated after CAR-T therapy had lost TAA expression (151, 175). Therefore, selecting patients for treatment depending on their percentage of expression is essential since high antigen expressing cells are preferentially killed within tumors meaning that lower antigen expression increases the risk for antigen escape (174).

Unlike TCR which benefits from an activation amplification system allowing them to recognize very low levels of antigens, CAR-T cells depend on a higher threshold for antigen density with low TAA densities limiting CAR-T cell activation (55, 176). Countering this issue with higher CAR expression is useful to a certain extent, as too high CAR densities lead to antigen independent activation and CAR-T cell exhaustion. At the same time, excessive antigen affinity of CAR increases the risk of on-target off-tumor toxicity. Severe toxicity stemming from on-target off-tumor cytotoxicity was reported in several clinical trials where CAIX targeting CAR-T cells infiltrated the antigen expressing bile-ducts causing grade 2 to 4 hepatotoxicity even at the lowest used treatment doses, as well as the severe respiratory toxicity in the trial assessing CEACAM5 targeting CAR-T cells which led to trial closure (141, 148).

Regarding CAR affinity, it seems that a good balance between activation by overexpressed tumor-associated antigen while avoiding activation by lower-level expression in normal tissues is more likely to be achieved in the range of Kd 10^-6^ – 10^-7^ M which is the natural affinity range of TCR (176). Another mechanism of resistance to therapy related to antigen heterogeneity besides antigen escape was discovered using tumor-derived organoids showing that antigen-negative tumor cells form shield-like structures protecting the high-expressing cells. Additionally, the authors of this study proposed a saturation mechanism for CAR-T cell therapies in solid tumors showing that effector cells which do not act to kill cancer cells, termed “free CAR-T cells” increase with higher therapeutic doses, leading to increased risks of side-effects (177).

### 4.6 The influence of microbiota in CAR-T cell therapy

Gut microbiota has been studied in various topics during the last decades, including autoimmunity, metabolic disorders, cardiovascular disease, neurodegenerative disorders and even in cancer. Gut microbiota has a critical role in immune regulation and could influence the outcome of antitumor therapies (178).

The role of gut microbiota in CAR-T cell therapies was evaluated by several groups, in retrospective studies, which are of high importance as the data suggests that the response to therapy and the toxicity of CAR-T cell therapy have a clear connection with microbiota. Smith et al. evaluated patients with R/R B-ALL and LBCL that received anti CD19 CAR-T cells using CD28 and 4-1BB costimulatory CAR-T cells, showing that the patients that received broad-spectrum of anaerobe-targeting antibiotics correspond with a decreased alpha diversity and the exposure to the antibiotic cure was correlated with reduced progression-free survival, overall survival and in the case of lymphoma patients, ICANS had higher incidence in those that received antibiotics (179).

The presence of *Bifidobacterium longum* and the peptidoglycan synthesis was strongly correlated with a long-term survival and response to therapy. Furthermore, it was highlighted that the presence of *Akkermansia muciniphila* could be potentially responsible for a better quality of the final CAR-T product as the CD3+ and CD4+ T cells count were favorable for generating a good quality product (180).

Hu et al. presented the case of multiple myeloma patients that have different gut microbiota patterns who achieved CR after anti-BCMA CAR-T cells. The research highlights different amino acid metabolism pathways enriched in responders versus nonresponders, with *Bifidobacterium* marked as enriched in CR patients and being associated with CRS (181).

The first observation of the relationship between gut microbiome and CAR-T cell therapy was made by Kuczma et al, who evaluated the anti-CD19 CAR-T cells in murine models. The study showed that the administration of a broad-spectrum antibiotics therapy was responsible for the alteration of the gut microbiome and was associated with a prolonged persistence of the CAR-T cells (182). While, on the other hand, Uribe-Hernadez et al. showed that vancomycin therapy administered in immunocompetent mice after receiving CAR-T cells experienced better lymphoma control, as the use of vancomycin enriched endogenous CD8+ T cells and Cd11+CD103+ dendritic cells (183).

Based on these findings and considering that microbiota has a key role in immune modulation, many therapeutic strategies have been developed to adjust microbiota activity to boost the antitumor effects of different immunotherapies: adjustment of antimicrobial therapy, diet, prebiotics, probiotics and fecal microbiota transplantation (178).

The gut microbiota has demonstrated considerable effects on cancer treatment, and immune functions. Initial findings indicate their possible connections in changing the effects of CAR T cell therapies, but the exact mechanisms have yet to be thoroughly explained. We have highlighted several potential therapeutic avenues to improve the performance of engineered T cells and improve the treatment of patients receiving CAR T therapy by utilizing the gut microbiota. Clinical trials are necessary to evaluate the possibility of these approaches and to achieve consistent improved outcomes.

## 5 Strategies to overcome the problems

### 5.1 Memory cell paradigm

Despite the initial success of immunotherapy with CAR-T cells in hematologic malignancies, high relapse rates and resistance remain major limitations that urgently need to be addressed. Although the exact mechanism is not yet clear, recent studies have shown that CAR-T cell exhaustion is closely related to epigenetic regulations such as gene modification, DNA methylation and histone acetylation (184–186). As previously described, HDAC inhibitors can significantly enhance the antitumor efficacy of T cells, but only in recent years have the effects of such a combination with CAR-T cells on therapeutic outcome been investigated in preclinical and limited clinical studies.

In addition to hematologic malignancies, some solid tumors that are generally more resistant to CAR-T cell therapy, mainly due to the immunosuppressive tumor microenvironment and antigen escape mechanism, have been shown to be more susceptible to CAR-T cells when HDACi is added to the treatment. The pan-HDACi vorinostat was able to increase the cytotoxic activity of CAR-T cells targeting the B7-H3 antigen in several solid tumor cell lines by increasing the expression of B7-H3 on the cell surface and downregulating immunosuppressive signaling pathways (187). Panobinostat resulted in substantial suppression of Her2+ pancreatic tumors in mice when co-administered with Her2-gp100 dual specific CAR-T cells and a vaccine that activates CAR-T cells by inducing apoptosis and memory cell formation (188). In a more recent attempt to improve CAR-T immunotherapy in pancreatic cancer, Zhang and coworkers incorporated short hairpin RNA (shRNA) sequences targeting HDAC11 into the NKG2D (Natural killer group 2 D receptor)-targeted CAR-T cells (they termed them sh-NKG2D-CAR) (189). *In vitro* studies on PC-3 and DU-145 cells showed that downregulation of HDAC11 by sh-NKG2D-CAR resulted in enhanced cytotoxicity compared to conventional CAR-T cells, which was attributed to enhanced T-cell activation and degranulation capacity as well as increased expression of Granzyme B (GzmB) and IFN. Sh-NKG2D-CAR were also able to promote proliferation and differentiation of CAR -T cells into memory T cells while reducing depletion, as demonstrated *in vitro* and in the pancreatic cancer xenograft model in mice. These reports provide a reliable basis for further clinical evaluation of CAR -T cell therapy in combination with HDAC inhibition as a promising strategy to increase efficacy and overcome resistance to CAR -T cell therapy in malignant B-cell tumors and some solid tumors. However, HDACi could induce DNA damage in both normal and cancer cells. Fortunately, normal cells could repair the HDACi induced DNA damage, which can explain the therapeutic window observed in clinical practice. This off target effect could be controlled as in the case of demethylating agents, by following a standard regimen, in cycles, allowing the normal cells to recover, while the tumor cells which grow faster and have intense metabolism, will still be affected by HDACi (190, 191).

The cornerstone of the CAR T-cell production process relies on the most effective T-cell product. Several strategies can be employed to overcome resistance in CAR T-cell therapy related to the memory cell paradigm, focusing on enhancing memory T-cell generation, maintenance, and function. Central memory T cells and stem cell memory T cells are associated with better clinical outcomes in CAR T-cell therapy. These subsets of T-cells possess the ability to self-renew and differentiate into effector cells upon encountering an antigen, offering the potential for long-lasting anti-tumor responses (192).

The choice of costimulatory domains in CAR design significantly impacts the differentiation and persistence of memory T-cells (193, 194). Several domains have been described in CAR T-cell products, but CD28 and 4-1BB are used in most clinical trials, and current CAR T-cells approved by the FDA contain one of these costimulatory domains. It was found that 4-1BB costimulation is more likely to lead to the new generation of central memory phenotype T cells with better proliferation, survival, cytokine secretion ability, and higher persistence than CD28 costimulation. In turn, CD28 promotes high cytotoxic activity and an effector-like phenotype (193). Combining 4-1BB and CD28 can enhance CAR T-cell activity, improve the central memory phenotype, boost proliferation, and increase recruitment of lymphocyte-specific protein-tyrosine kinase to the CAR (38).

Selecting memory-like characteristics in T cells used for CAR T-cell manufacturing can improve outcomes. It was observed that a memory profile in CD8+ CAR T cells, marked by elevated CCR7, CD27, and SELL expression in the infusion product, has been associated with complete response (CR). In contrast, patients with a more exhausted CD8+ CAR T cell phenotype tend to show a poorer early molecular response, as indicated by tumor-derived cell-free DNA levels in plasma (195). Also, central memory phenotype CAR T cells have been associated with higher *in vivo* and *in vitro* activity than effector memory phenotype T-cells (196). Another evidence is that an equal CD4:CD8 ratio in the CAR T cell product correlates with better outcomes (197–199). Implementing a 1:1 ratio of both CD4:CD8 Chimeric Antigen Receptor (CAR) T cells can improve outcomes. This consideration is implemented in the manufacturing workflow where CD4+ and CD8+ T cells are co-cultured, and ratios are defined during the initial culture stage. This strategy has been observed to promote the expansion and activity of CD8+ CAR T cells. The CD4+ cells serve to maximize proliferation and support the maintenance of a functional CD8+ T cell phenotype, which is essential for anti-tumor activity, during the initiation of culture. Coculturing creates a population of CD4+ and CD8+ T cells at a 1:1 ratio, which improves upon the expansion, phenotype, and *in vivo* anti-tumor activity of CAR T cells compared to isolated cultures of CD8+ T cells. Typically, the manufacturing process is to select and enrich CD4+ and CD8+ T cells simultaneously, and then co-culture them in a specified ratio. This is a practical method since it reduces the manufacturing process, and if done properly, will lead to a balanced CAR T cell product. CD4+ cells have a beneficial function on CD8+ through both cytokines signaling as well as cell contact, through mechanisms including CD40L-CD40 and CD70-CD27 (145, 200). Additionally, Galli et al. found that a lower CD4/CD8 ratio in the infused CAR T cell product was associated with better clinical responses at 3 and 6 months post-treatment (201). The controlled ratio of CD4/CD8 ratio for CAR T cell manufacturing has several limitations such as the high complexity in manufacturing the product, as separate cultures of CD4 and CD8 positive cells do complicate the process and implies additional resources and time. Coculturing at different ratios can simplify the manufacturing process.

Producing CAR T cells with a stem central memory phenotype can also be an option to improve outcomes once these cells have a more fit metabolism with more vigorous killing activity and persistence (202).

Altering the metabolic pathways of CAR T cells can foster the emergence of a memory phenotype. FOXO1 is a key regulator for memory programming in CAR T cells, boosting their stemness, metabolic health, and effectiveness (203–205). At the same time, the NOTCH-FOXM1 pathway contributes to the formation of stem cell memory-like CAR T cells (206, 207). Additionally, overexpression of PRODH2 in CAR T cells reprograms proline metabolism, promoting mitochondrial proliferation and oxidative phosphorylation, reducing glycolysis, and increasing the generation of memory cell phenotype CAR T cells (208, 209). Also, inhibition of IDH2 with small-molecule inhibitors leads to an increase in glutamine oxidation and inhibits KDM5-dependent H3K4 demethylation, increasing the ability of CAR-T cells to differentiate into memory cells (210). Thus, reducing glycolysis and enhancing glutaminolysis and polyamine synthesis are potential strategies to improve CAR T-cells’ persistence and immune characteristics (211). Transient rest can restore functionality in exhausted CAR-T cells via epigenetic remodeling. This can be done by disrupting TET2, which promotes the formation of memory cells that results in increased efficacy (212). Knocking out DNA methyltransferase 3 alpha (DNMT3A) retains a stem-like phenotype, preventing exhaustion and enhancing antitumor activity (213).

For stem central memory phenotype CAR T cell production, a preselection of naïve and stem memory T cells can enhance the CAR T cell antitumor responses and persistence, with the cells exhibiting an increased expansion rate. These being translated into better long-term efficacy (214). Another way to generate these stem central memory phenotype CAR T cells is to incorporate the membrane-bound IL-15, as Hurton et al. mentioned (215). Coexpressing CAR with membrane bound chimeric IL-15 can promote the development of T cells with a stem central memory, this approach enhancing the persistence and antitumor activity of the CAR T cells. The manufacturing process limitations for these CAR T cells include the complexity of cell selection and expansion as preselection of naïve and stem memory T cells is technically challenging; moreover, the growth media needs specific concentrations and ratios of cytokines and other growth factors, and then the TME challenges can impair the function of these CAR T cells. The main issue with the manufacturing process for these naïve and stem central memory phenotype CAR T cells is the variation between batches, as the T cell quality may be different for one donor to another (216–218).

Metabolic interventions can be feasibly implemented in clinical-grade CAR-T cell manufacturing, while several strategies have been tested to enhance CAR-T cell metabolic fitness and their antitumor efficacy (219). Modulating *ex-vivo* culture conditions such as cytokine supplementation, nutrient composition and the use of metabolic pathway activators or inhibitors, all during the manufacturing process to produce less differentiated memory-like T-cell phenotypes with improved persistency (220). These changes should be integrated into current GMP workflows during expansion and activation phases. Any added agents should pass the regulatory compliance and safety, all changes should be compatible with the automated close-system bioreactors and should not induce variability in products. In the end, the quality control should be passed without any unintended effects on T-cell phenotype and functionality (221). The current implementation of metabolic interventions for next-generation CAR-T cells investigates the modulation of cytokine cocktails, modulation of glucose and amino acid concentrations or the transient exposure to metabolic modulators during the expansion phase.

### 5.2 Short-lived effector cell paradigm

The short-lived effector cell paradigm involves differentiating T cells into effector cells that can rapidly respond and eliminate tumor cells. While these cells are crucial for immediate tumor control, they have a limited lifespan and may not provide long-term protection. Indeed, T-cell exhaustion, characterized by the loss of effector functions, is a significant limitation in CAR Tcell therapy (222, 223).

Disrupting checkpoint signal pathways is a common strategy to reduce CAR-T cell dysfunction and restore their efficacy. PD-1 blockade can increase memory phenotype, reduce exhaustion, and induce durable responses of CAR-T cells (224, 225). The combination of PD-1 antibody checkpoint blockade and CAR-T cells demonstrated enhanced effectiveness of CAR-T cell therapy in both preclinical and clinical studies (226). For example, A Phase I clinical trial demonstrated that anti-mesothelin CAR-T cells, combined with the anti-PD-1 agent pembrolizumab, exhibited therapeutic effects in patients suffering from malignant pleural disease (227). In another study, CAR-T cells armed with autocrine PD-L1 scFv antibody reversed exhaustion and enhanced anti-tumor immune response in solid tumors and hematologic malignancies by blocking the PD-1/PD-L1 signaling (228). CRISPR technology can also be used to disrupt checkpoint pathways. A study showed the preliminary feasibility and safety of CRISPR-engineered CAR-T cells with PD-1 disruption and suggested that the natural TCR plays an important role in the persistence of CAR-T cells when treating solid tumors (229). Additionally, researchers have worked on engineering CARs that arm cytokines or express cytokine receptors, swapping inhibitory domains for activation domains in PD-1 or TGF-β as switch receptors, as well as deleting negative regulators in T cells or overexpressing factors that enhance T cell function (230). Regarding CTLA4, its deficiency improved proliferation and anti-tumor efficacy in preclinical models of leukemia and myeloma, rescuing the function of T cells from patients with leukemia who previously failed CAR-T cell treatment (231).

Specific cytokines can promote memory cell formation and persistence. IL-15 can enhance CAR-T cell activity by reducing mTORC1 and preserving stem cell memory phenotype with better metabolic fitness. This results in superior vivo antitumor activity, creating a pathway to improve future adoptive T-cell therapies (232, 233). IL-15 also can protect NKT cells from inhibition by tumor-associated macrophages and enhance anti-metastatic activity (234). Co-expression of IL-4/IL-15 based inverted cytokine receptor in CAR-T cells overcomes IL-4-mediated immunosuppression in solid tumors (235). The expression of IL-7 and CCL19 in CAR-T cells enhances immune cell infiltration and supports the survival of CAR-T cells within tumors (236). Furthermore, tumor-targeted CAR T cells can secrete IL-12 and IL-18 to eliminate ovarian and other tumors effectively (237, 238).

Metabolic interventions can also enhance the effector function of CAR T cells (239, 240). For example, targeting the glycolytic metabolism and polyamine/hypusine axis can control the generation of CD8+ tissue-resident memory T cells (241). Additionally, NAD+ supplements can potentiate tumor-killing function by rescuing defective TUB-mediated NAMPT transcription in tumor-infiltrated T cells (242). Enhancing fatty acid catabolism can increase the efficacy of immunotherapy by improving the CD8+ tumor-infiltrating T lymphocytes’ ability to slow tumor progression (243). Another strategy is integrating stearoyl-CoA desaturase 1 (SCD1) inhibitors with CAR-T cell therapy to improve the antitumor effects. SCD1 inhibitors block the conversion of saturated fatty acids, including palmitic and stearic acids, into mono-unsaturated fatty acids via ACAT1-dependent reduction of esterified cholesterol. Therefore, the SCD1-ACAT1 axis regulates effector functions of CD8+ T cells, and SCD1 inhibitors and ACAT1 inhibitors are attractive drugs for cancer immunotherapy (244).

The role of HDACs in T cells has been extensively studied in recent years, and many of them have been shown to be important for T cell development and function (245, 246). Shen and Pili (2012) (247) demonstrated that Class I HDAC inhibitors can specifically target Treg cells and thereby disrupt immune tolerance in cancer. Their study showed that the HDAC1 inhibitor entinostat suppresses Treg function, thereby increasing antitumor activity and immunotherapy efficacy in mouse models of renal cell carcinoma and prostate cancer (248). The study showed that entinostat represses Foxp3 expression at either the transcriptional or post-transcriptional level, resulting in a reduction in Foxp3 protein levels and impaired suppressive function in Treg populations, while the total number of peripheral Treg cells remains unaffected (248). The mechanism by which entinostat exerts its effect on Treg cells is primarily mediated by Signal Transducer and Activator of Transcription 3 (STAT3). STAT3 forms a complex with HDACs 1 and 3, which leads to hyperacetylation of STAT3 (249). Entinostat has been confirmed to specifically target STAT3, triggering its acetylation and pathway activation, leading to suppression of Foxp3 gene expression and reduced inhibitory function of Tregs (249). HDAC3 also plays a crucial role in modulating the suppressive function of Treg cells. Conditional deletion of HDAC3 in Foxp3^+^ Treg cells disrupts both the development of Treg cells and their suppressive function (250). In addition, the study by Wang and co-authors (2018) shows that conditional deletion of HDAC8 in Foxp3^+^ Treg cells or the use of HDAC8 inhibitors impairs Treg function and promotes anti-tumor immunity (251). SIRT2 moderately suppressed Foxp3 expression as well as the immunosuppressive function of Tregs (252).

On the other hand, Trichostatin A, a pan-HDAC inhibitor, enhances the differentiation and suppressive function of Treg (247, 253–255). Further studies are needed for each HDAC isoform and their effects on Treg cells. Overall, these data suggest that HDAC enzymes affect the immunosuppressive function of Treg cells in tumor microenvironment.

### 5.3 Overcoming trafficking

A significant obstacle for CAR T-cell therapy in solid tumors is the poor trafficking of T cells to the tumor sites. This can happen not only because the immunosuppressive TME can hinder CAR-T cell activity but also because tumor stroma and physical barriers limit the mobility and penetration of CAR T cells (256–258).

Directly administering CAR T-cells into the tumor can bypass the need for systemic trafficking, increase their concentration at the tumor site, and mitigate off-tumor toxicities (259, 260). Local delivery can result in an earlier and increased accumulation of CAR-T cells within the tumor and induce systemic and long-lasting anti-tumor immunity (256–259). For example, pre-clinical models have demonstrated the superior therapeutic efficacy of intraventricular injection of CAR-T cells targeting HER2 and IL13Rα2 in breast cancer brain metastases and glioblastoma, respectively (261, 262). Likewise, preclinical models showed superior CAR-T cell treatment of malignant pleural mesothelioma through intrapleural injection (107). Moreover, a transdermal porous microneedle patch was observed to allow the intra-tumoral penetration of CAR-T cells and enhance their infiltration compared to direct intra-tumoral injection in solid tumors (263).

Engineering CAR T-cells to express chemokine receptors can enhance migration to the tumor. Low radiation doses and phosphoramide can modify ligands secreted by the TME, augmenting cell trafficking by inducing the expression of CXCR4 and CXCL-12, blocking inhibitory cytokines and receptors, and reducing the expression of the endothelin B receptor (155, 264, 265). Experimental studies in murine models have shown encouraging results on pancreatic cancer through the negative regulation of pro-tumor cytokines (266). Some tumors can restrict T cell infiltration by reducing the expression of T cell-recruiting chemokines or adhesion molecules essential for extravasation. This can be found in brain, breast, plural, and liver cancers (267, 268).

Designing CAR-T cells to secrete matrix-degrading enzymes can disrupt physical barriers in solid tumors and improve infiltration (269). This can be achieved by engineering CAR-T cells to secrete the heparanase enzyme, which can degrade the tumor matrix and overcome tissue barriers and targeting CAR-T cells to fibroblast activation protein to remove stromal cells (270–272). Other strategies were found to increase trafficking, such as disrupting the “sugar coat” by designing molecules that can break the sugar shield that tumors use to resist CAR -T cell attack (273), the combination of immune therapy with oncolytic viruses with effective tumor debulking by destroying the molecular shield used by some solid tumors to escape the immune system attack (274, 275), and using nanobody-based CAR-T cells such as PD-1/CTLA-4- antibodies secreting CAR-T cells (276).

### 5.4 Overcoming tumor heterogeneity

Tumor heterogeneity, the variation in antigen expression within a tumor, is another challenge for CAR -T cell therapy (192, 224, 258). Tumor cells can downregulate or lose the target antigen, leading to resistance, such as on biallelic loss of BCMA has been observed as a resistance mechanism to CAR -T cell therapy and EGFRvIII-directed CAR -T cells mediating antigen loss and inducing adaptive resistance (151, 277). Additionally, the varied and limited antigens found in solid tumors, as opposed to those in liquid tumors, create a significant challenge for successful CAR -T cell therapy (258).

One approach to mitigating antigen escapes phenomena commonly associated with CAR-T cell therapy involves combinatorial strategies, such as sequential or combination treatments involving different CAR-T cell products that concurrently target multiple antigens. This strategy has already proven to be both clinically safe and effective in DLBCL (diffuse large B-cell lymphoma), and it could also offer a promising approach for treating solid tumors (278, 279). Another approach involves creating multitarget CAR-T cells, which can be done by integrating two different CAR constructs into T cells or using bi-specific or Tandem CAR-T cells. For example, in breast cancer, bi-CAR-T cells targeting ErbB2 and MUC1 *in vitro*, showed efficient antitumor activity (280). In glioblastoma, combinational targeting offsets antigen escape and enhances effector functions of adoptively transferred T cells, namely T cells coexpressing HER2 and IL-13Rα2-CARs (281). Tandem CAR -T cells feature a paired arrangement of two single-chain variable fragments (scFv). Research revealed that a tandem configuration of IL13 and EphA2 scFv demonstrated that the IL13-anti-EphA2 TanCAR showed significantly enhanced anti-tumor efficacy compared to single CAR-T cells, in both *in vitro* and *in vivo* settings (282).

Using synthetic Notch (SynNOTCH) receptors to control CAR -T cell activity can overcome challenges of specificity, heterogeneity, and persistence challenges. With this approach, the SynNOTCH receptor is activated by one tumor antigen and triggers the expression of a CAR against a second tumor antigen. Using this strategy, CART cells are only active and kill when both antigens are present (283). An alternative approach to antigen escape has been successfully demonstrated in AML models with CD70 loss by engineering CD70-targeting CAR-T cells to secrete a CD3/CD33 bispecific T cell engager. This strategy enables the cells to effectively overcome escape mechanisms involving either CD70 or CD33 (284).

Intrinsic tumor antigen expression and intratumoral heterogeneity can be rendered irrelevant by tagging tumors with small molecules such as FITC, which act as surrogate targets in a universal manner. A key benefit of this approach is that CAR-T cells can target both tumor cells and tumor-infiltrating cells such as MDSCs and tumor associated macrophages (TAMs) indiscriminately, while also priming endogenous cell-mediated immunity. However, a significant limitation is that tumors must be tagged via intratumoral injection, restricting this strategy to large, accessible tumors (285).

Modular CAR-T cells represent a remarkable concept that enables fine-tuning of therapeutic functions to address tumor antigen heterogeneity. This technology separates CARs into interchangeable, interlocking units, allowing engineered T cells to become universal and function with various target antigens through the simple addition of compatible Fvs. One notable example is the split, universal, and programmable (SUPRA) system, which uses leucine zippers to connect CAR modules, tailor binding affinities, and introduce logic gates to both enhance sensitivity in heterogeneous tumors and reduce on-target, off-tumor toxicity (286).

Modular CAR-T cells are poised to expand the scope of T cell redirection, as multiple similar platforms developed by commercial companies—such as SparX-ARC-T from Arcellx and OmniCAR from Prescient Therapeutics—broaden the repertoire of antigen recognition domains and enable enhanced CAR-T fine-tuning capabilities (287).

Additionally, CAR -T cells can be combined with treatments that boost Fas expression on tumor cells, like Smac mimetics or BCL-2/xL inhibitors. This approach would circumvent tumor heterogeneity and tumor cells’ resistance to CAR -T cell elimination (46). Switching CAR-T cells on or off can also control activation and inhibition. For example, using a bifunctional small “switch” molecule composed of folate and fluorescein isothiocyanate allowed CAR-T cells to identify tumor cells overexpressing folate receptors specifically (288). Furthermore, employing suicide genes or activating antibody-mediated killing can inhibit CAR-T cell functionality. Specifically, integrating the inducible caspase 9 system into CAR-T cells triggers apoptosis, resulting in reduced CAR-T cell activity (289).

Targeting components of the tumor microenvironment, such as fibroblast activation protein (FAP) on stromal cells, can indirectly affect tumor growth and survival and overcome tumor heterogeneity. FAP is a protease produced by cancer-associated fibroblasts (CAFs) and is involved in the remodeling of the tumor extracellular matrix (ECM). Research has shown that the adoptive transfer of FAP-CAR -T cells diminishes tumor growth in a FAP-dependent manner and can eliminate stromal cells, evident in several solid tumors, including mesothelioma, lung cancer, and pancreatic cancer, demonstrating antitumor activity in preclinical models (272, 290–292). CAR -T cells can be engineered to release cytokines that modify the tumor stroma, enhancing their therapeutic effects. These engineered cells, sometimes called “armored” CAR -T cells or TRUCKs (T-cells Redirected for Universal Cytokine Killing), can express various cytokines, interleukins, pro-inflammatory ligands, or chemokines to counteract the immunosuppressive environment of solid tumors (293). Many cytokines, including IL-2, IL-4, IL-7, IL-8, IL-9, IL-10, IL-12, IL-15, IL-18, IL-21, IL- 23 are being investigated for their ability to enhance CAR-T activation and persistence (42). For instance, CAR-T cells directed at the extracellular domain MUC, designed to secrete IL-12, demonstrated improved efficacy in preclinical ovarian cancer models (237). Additionally, CAR-T cells engineered to release IL-18 successfully modulated the tumor microenvironment, significantly enhancing their *in vivo* expansion, persistence, and survival (38).

Many advances in CAR-T cell design offer solutions to isolated challenges posed by solid tumors. However, clinical efficacy of these therapies may lie in integrating these models into intelligent, environment-sensing CAR-T cells using logic gates and modular CARs, which can adapt and regulate activity in response to tumor-specific cues to maintain efficacy amid dynamic changes such as antigen density variations, hypoxia, and suppressive cell pressure. In parallel, the field must recognize that effective therapy also requires functional trafficking—ensuring that CAR-T cells not only reach but also survive and operate within tumors (294, 295). These biologically tuned CARs should be co-developed with adjunctive strategies such as localized immunomodulation, matrix remodeling agents, or oncolytic viruses to dismantle the hostile tumor stroma and create a receptive environment for T cell action. Such integrative designs will likely be essential to achieve durable responses in solid tumors.

## 6 Successful trials

Despite significant challenges in the field of adoptive cell therapies for solid tumors, several successful trials bring hope that this approach might someday improve the outlook of these patients. A phase I clinical trial showed remarkable responses using Claudin18.2 (CLDN18.2) second generation CAR-T for the treatment of CLDN18.2 positive gastrointestinal cancers. This trial included 37 patients and led to radiographic tumor reduction in 83.3% of patients, with an overall response of 48.6% according to RECIST criteria. While the median persistence of CAR-T cells was 28 days, it ranged from 14 to 203 days. As expected, responders showed higher peak expansion, with peak values over 2-fold higher than non-responders which seems to be more characteristic of more naïve CAR-T subsets. Additionally, a composite indicator of both persistence and peak expansion, the CAR-T cell AUC_last_ as determined until the last measurable value seems to be more relevant for efficacy and positively correlated with PFS. Although it was shown that 75% of patients developed anti-drug antibodies, it did not influence response to treatment. Perhaps contributing to the remarkable response rates, repeated biopsies following CAR-T infusion did not show TAA downregulation (296).

More recently, an Italian phase I-II study using a 3^rd^ generation CAR-T cell therapy engineered to express the iCAS9 suicide gene achieved exceptional responses in pediatric refractory neuroblastoma, with one third of patients achieving complete response. The recommended dose selected after assessment of dose-limiting toxicity was 10x10^6^/kg, which is very similar to the doses used in the CLDN18.2 trial. Out of the 27 patients treated, 9 and 8 patients achieved CR and PR respectively. An unusual occurrence is that three of the patients with partial responses show long term persistence of response, still maintained at cutoff. In this trial, one patient developed severe CRS in which rimiducid was effectively used to rapidly reduce circulating CAR-T cell levels. Remarkably, after 6 weeks, the CAR-T cells re-expanded and the patient was one of the nine achieving CR. Additionally, CAR-T cells preserved their iCas9 mediated sensitivity to rimiducid after re-expansion. In relapsing patients, however, despite preserved tumor antigen expression, CAR-T cells do not re-expand; however, one patient achieved a second CR after repeat infusion. In this trial, high-burden disease was the most important risk factor, and none of these patients were alive at the 3-year time point (297).

The shared features of these successful trials (**Table 1**) might be highly indicative of what will prove to be the future of CAR-T cell therapy in solid malignancies. Therapeutic doses used in both trials seem to be similar when accounting for the differences in weight between pediatric patients and adults, and the CAR-T subtype composition of infusion appears to be determinant of responses. Additionally, the preservation of antigen expressions including in relapse may indicate that downregulation might be antigen specific and that better understanding of what leads to this property might allow mitigation of antigen-escape through careful target selection.

**Table 1:** Response rates in phase I and I/II trials of CAR-T cell therapies in solid cancers.

Clinical trial and sources	Trial Phase	CAR-T Gen	Target Cancer	Target Antigen	Costim. Domain	Best radiologic response	Observations
NCT03874897 (298, 299)	I	2^nd^	CLDN18.2 positive GI tumors	CLDN18.2	CD28	37.8% ORR 75.5% DCR	CLDN18.2 IHC expression >40% No dose limiting toxicities
NCT04581473 (300)	II	2^nd^	CLDN18.2 positive pancreatic cancer	CLDN18.2	CD28	16.7% ORR 70.8% DCR	CA19-9 level reduction in the majority of patients mPFS and mOS benefit in patients achieving PR/SD
NCT04196413 (301, 302)	I	2^nd^	H3K27M-mutated diffuse midline gliomas	GD2	4-1BB	N.R.	Intravenous and intracerebroventricular infusions serial infusions 83% of patients showed clinical and/or radiograhic benefit
NCT02761915 (303)	I	2^nd^	Pediatric neuroblastoma	GD2	CD28	0% ORR	Regression of soft tissue and bone marrow disease in 25% of patients
NCT01822652 (146)	I	3rd	GD2 positive solid cancers	GD2	CD28, OX-40	41.6% ORR 58.3% DCR	Used in combination with BRAF/MEK inhibitor therapy in metastatic melanoma Increased CAR-T expansion and persistence with protocols for CAR-T enriched for central-memory-like, CCR7 and CD62L-expressing cells
NCT03373097 (297)	I/II	3^rd^	Relapsed/refractory neuroblastoma	GD2	CD28, 4-1BB + iCas9	33.3% CR 63% ORR 83% DCR	Successful elimination of GD2-CART by activation of suicide gene in 1 patient with severe CRS
NCT04483778 (304)	I	3rd	Non-CNS B7-H3 positive solid tumors	B7-H3	CD28, 4-1BB	11% ORR 44% DCR	Systemic administration
NCT04185038 (305)	I	2^nd^	Diffuse intrinsic pontine glioma (DIPG)	B7-H3	4-1BB	6% ORR 89% DCR	Intracerebroventricular serial infusions mOS 19.8mo is higher than historical mOS
NCT01869166 (306, 307)	I	2nd	EGFR-positive advanced unresectable, relapsed/metastatic cancers	EGFR	4-1BB	In BTC ORR 5.8% DCR 64.7% In PC ORR 28.5% DCR 85.7%	EGFR IHC expression >50% Grade I/II on-target off-tumor toxicity 1-3 repeated infusions
NCT02209376 (151)	I	2^nd^	EGFRvIII positive glioblastoma	EGFRvIII	4-1BB	ORR 0% DCR 10%	No on-target off-tumor toxicity Stable disease > 18mo. in one patient
NCT03618381 (308)	I	2^nd^	Non-CNS EGFR positive solid tumors	EGFR806	4-1BB	N.R.	DLT hepatotoxicity in 1/10 patients 20% of patients showed mixed response
NCT02414269 (227)	I	2nd	Malignant pleural cancers	Mesothelin	CD28	8.6% ORR 56.5% DCR	Mesothelin IHC expression >10% 11% CR achieved in patients who also received Pembrolizumab
NCT01935843 (309)	I	2^nd^	advanced BTC and PC	HER2	CD28	9% ORR 54.5% DCR	HER2 IHC expression >50% 1/11 patients grade III hepatotoxicity
NCT00902044 (310)	I	2^nd^	HER2-positive sarcomas	HER2	CD28	21% ORR 50% DCR	
NCT01212887 (148)	I	1^st^	Carcinoembryonic (CEA) positive tumors	CEACAM5	none	0% ORR 50% DCR	CEA positivity defined by IHC or CEA > 50 μg/L Acute respiratory toxicity attributed to on-target off-tumor toxicity
NCT03089203 (156)	I	Armored CAR	Metastatic castrate resistant prostate cancer	PSMA	4-1BB	0% ORR 38.5% DCR	PSMA IHC expression >10% >30% PSA reduction in 23% of patients Uses dominant-Negative TGF-β Receptor CAR-T cells Evidence of tumor regression in 1 patient
NCT02706392 (311)	I	2^nd^	ROR1 positive triple-negative breast cancer or non–small cell lung cancer	ROR1	4-1BB	5.5% ORR 94% DCR	ROR1 IHC expression >20% After 6 mo., all patients had progressed
NCT05103631 and NCT04377932 (312)	I	IL-15 armored	GPC3 positive solid tumors	GPC3	4-1BB	33% ORR 66% DCR	Increased CRS incidence associated with IL-15 armored CAR-T

IHC, immunohistochemistry; N.R., not reported; CLDN18.2, claudin18.2; GD2, disialoganglioside GD2; EGFR, Epidermal growth factor receptor; EGFRvIII, EGFR variant III; HER2, human epidermal growth factor receptor 2; CEACAM5, carcinoembryonic antigen; PSMA, prostate-specific membrane antigen; ROR1, Receptor tyrosine kinase-like Orphan Receptor 1; GPC3, Glypican-3; BTC, biliary tract cancers; PC, pancreatic cancers; N.R., not reported; ORR, overall response rate; DCR, disease control rate (complete response + partial response + stable disease)

## 7 Conclusions

CAR-T cell therapies have the potential to become the upfront treatment for both hematologic and solid malignancies. Still, for solid tumors, clinical applications face several roadblocks which are difficult to foresee in preclinical studies. The architectural complexity and heterogeneity of tumors creates physical and immunological barriers leading to poor trafficking and infiltration of CAR-T cells as well as an immunosuppressive TME which limits the antitumoral potential of current CAR-T cell therapies.

Success in overcoming these challenges rests on several pillars: characterizing and understanding mechanisms of resistance towards CAR-T cell therapies, accurately modeling the components of the CAR-T–tumor interface, and designing predictive models of clinical efficacy.

The current *in vitro* and *in vivo* models often fail to fully recapitulate the dynamic and immunologically complex environment of human tumors, leading to discrepancies between preclinical promise and clinical efficacy. Advanced experimental models such as patient-derived organotypic spheroids (PDOS) and humanized mice models provide more accurate platforms which promise to bridge this gap and allow testing of new CAR-T cell designs and strategies to find solutions for tumor resistance to treatment.

The costs for the preclinical setup would increase if testing the CAR-T cells on different organoid, humanized mice and by applying multi-omics approaches, moreover, many pipelines would need improvements, but all these investments and challenges will lead to better understanding and to a comprehensive overview of the next-generation CAR-T cells. Moreover, using such a variety of *in vitro* and *in vivo* models could offer a better prediction of the potential outcome, limiting the future negative outcomes which will come with extra costs to counter them in later stages of the clinical trials. The regulatory complexity will increase, while the benefits and outcomes are worth the investments and challenges during the preclinical setups.

Innovations in CAR design, such as armored CARs, TRUCKs, dual-targeting CARs, modular and logic-gated CARs are being developed to enhance persistence, trafficking, and functional adaptability of CAR-T cells in solid tumors. Additionally, refining the phenotype composition of CAR-T products to favor stem-like and memory T-cell subsets has shown promise in increasing durability and response rates and adjuvant therapies may be used to mitigate the immune suppressive effects of the TME and aid in overcoming tumor heterogeneity issues.

Despite these hurdles, there have been encouraging signs of clinical efficacy in solid tumors, perhaps owing to a synergy of effective conditioning, target antigen selection and CAR design. Such notable success seen in phase I trials for gastrointestinal cancers and pediatric neuroblastoma where patients achieved remarkable objective tumor responses underscore the feasibility of CAR-T cell therapies for solid tumors when optimally designed.

Ultimately, the future of CAR-T cell therapy in solid tumors lies in a comprehensive approach: coupling technological advancements in cell engineering with the continual refinement of preclinical models and translational strategies. Robust and iterative evaluation frameworks integrating transcriptomic, proteomic, and immunologic data will be crucial for rational CAR target selection and for overcoming the limitations posed by the solid tumor milieu. While the road ahead is complex, sustained multidisciplinary efforts hold the promise of unlocking the full therapeutic potential of CAR-T cells across a broader spectrum of cancers.
